# Designing for impact: a case study of UTHSC's research impact challenge

**DOI:** 10.5195/jmla.2025.2085

**Published:** 2025-01-14

**Authors:** Jess Newman McDonald, Annabelle L. Holt

**Affiliations:** 1 mcdo1364@umn.edu, University of Minnesota, MN; 2 annabelle.holt@uky.edu, University of Kentucky, KY

**Keywords:** Education Technology, Research Metrics, Library Instructions, LibGuides, Health Science Libraries, Research Data Management, Outreach

## Abstract

Prompted by increasing requests for assistance with research evaluation from faculty researchers and university leadership, faculty librarians at the University of Tennessee Health Science Center (UTHSC) launched an innovative Research Impact Challenge in 2023. This Challenge was inspired by the University of Michigan's model and tailored to the needs of health sciences researchers. This asynchronous event aimed to empower early-career researchers and faculty seeking promotion and tenure by enhancing their online scholarly presence and understanding of how scholarship is tracked and evaluated.

A team of diverse experts crafted an engaging learning experience through the strategic use of technology and design. Scribe slideshows and videos offered dynamic instruction, while written content and worksheets facilitated engagement and reflection. The Research Impact Challenge LibGuide, expertly designed with HTML and CSS, served as the central platform, ensuring intuitive navigation and easy access to resources (https://libguides.uthsc.edu/impactchallenge). User interface design prioritized simplicity and accessibility, accommodating diverse learning preferences and technical skills.

This innovative project addressed common challenges faced by researchers and demonstrated the impactful use of technology in creating an adaptable and inclusive educational experience. The Research Impact Challenge exemplifies how academic libraries can harness technology to foster scholarly growth and support research impact in the health sciences.

The Research Impact Challenge was an asynchronous 5-day instructional series that ran August 7–11, 2023. Daily “Challenge” activities were designed to help participants better understand and manage their online scholarly presence and the impact and reach of their research. The intended audience for these activities was health science researchers and their support personnel, including graduate students.

- Day 1: Claim or Enhance Your Online Presence
- Day 2: Understand and Locate Your Research Impact Metrics
- Day 3: Reach a Wider Audience with Open Access Publishing
- Day 4: Learn How and Why to Share Your Research Data
- Day 5: Stay Informed and Build Your Network

The 2023 Challenge team comprised eight faculty librarians, one communications and marketing specialist, and one Institutional Research staff member. Three library faculty worked on the initial SpringShare LibGuide design, including one HTML/CSS specialist. Subsequent edits to the LibGuide content were made by the project lead, including updates for year two of the challenge.

The technology utilized throughout the challenge included a custom LibGuide, videos, written instructions, and Scribes to users to engage with the content on each day of the challenge. The custom LibGuide, designed with HTML and CSS, served as the central platform to facilitate intuitive navigation and seamless access to resources. The interface design prioritized simplicity and accessibility, accommodating a wide range of learning preferences and technical skills. For each daily topic, the project team used collapsible accordion navigation tools or menus to display the tasks involved to complete each challenge. Each task included a combination of written instructions, videos, links, and Scribes. The team employed the free version of Scribe to develop step-by-step tutorials, enabling users to complete tasks asynchronously with ease (https://scribehow.com/).

In 2023 the challenge had 62 total registrations, including 40 faculty, 16 staff, and five students. The LibGuide received 352 views during the challenge week, August 7–11, 2023. At the end of the week a Qualtrics feedback survey was distributed to participants. Participation was encouraged by offering a “Certificate of Completion” upon finishing the survey. All respondents (19 total) were affiliated with UTHSC and the majority were faculty (73%). Of those that did not complete the challenge (21%), most indicated that they intend to finish it as they have time. Seventy-nine percent indicated that they were highly likely (7 or above out of 10) to recommend the challenge to a colleague. Impact Metrics, Scholarly Profiles, and Data Sharing were the most highly rated days, although all days received above-average ratings. Participants strongly agreed that the Challenge was “well organized” and of “sufficient quality.” Forty-two percent of respondents were surprised to learn that the Library had expertise on these topics, an additional 37% were somewhat surprised, indicating that the event was successful in promoting librarian expertise. Several participants, via this survey and personal email correspondence, expressed hope that the Challenge would remain available online for future use and dissemination.

For the second iteration of the Research Impact Challenge all content was created and updated in the LibGuide by the project lead. The challenge was again advertised and marketed by the communications specialist via various social media and internal communication channels. The 2024 challenge saw a slight drop in registrations, 57 total. The LibGuide received 185 views over the second challenge week, down from 352 the first year. This may be due to a variety of factors - a shorter lead up time for marketing in year two, the majority of the challenge content remained unchanged and thus there was no incentive for repeat participation, and prospective participants may have understood that registration was not required to access the content and that it would remain available after the challenge. Participants again received an anonymous link to a Qualtrics feedback survey with a new 2024 Certificate of Completion. The UT Health Science Center Library envisions that the challenge will be re-released annually with slight modifications and updates. As of Fall 2024 Library liaisons are in conversation with a College of Health Professions faculty member to include the challenge modules in their curriculum for the upcoming 2025 spring semester.

